# Characterization of High Molecular Weight Pneumococcal Conjugate by SEC-MALS and AF4-MALS

**DOI:** 10.3390/polym14183769

**Published:** 2022-09-09

**Authors:** James Z. Deng, Jason Lin, Michelle Chen, Catherine Lancaster, Ping Zhuang

**Affiliations:** 1 Vaccine Analytical Research & Development, Merck & Co., Inc., Rahway, NJ 07065, USA; 2 Wyatt Technology Corporation, Goleta, CA 93117, USA

**Keywords:** pneumococcal conjugate vaccine, protein polysaccharide conjugates, size-exclusion chromatography, multi-angle light scattering, asymmetrical flow field flow fractionation

## Abstract

Infections by Streptococcus pneumoniae can cause serious pneumococcal diseases and other medical complications among patients. Polysaccharide-based vaccines have been successfully developed as prophylactic agents against such deadly bacterial infections. In the 1980s, PNEUMOVAX^®^ 23 were introduced as the first pneumococcal polysaccharide vaccines (PPSV). Later, pneumococcal polysaccharides were conjugated to a carrier protein to improve immune responses. Pneumococcal conjugate vaccines (PCV) such as PREVNAR^®^ and VAXNEUVANCE™ have been developed. Of the more than 90 pneumococcal bacteria serotypes, serotype 1 (ST-1) and serotype 4 (ST-4) are the two main types that cause invasive pneumococcal diseases (IPD) that could lead to morbidity and mortality. Development of a novel multi-valent PCV against these serotypes requires extensive biophysical and biochemical characterizations of each monovalent conjugate (MVC) in the vaccine. To understand and characterize these high molecular weight (Mw) polysaccharide protein conjugates, we employed the multi-angle light scattering (MALS) technique coupled with size-exclusion chromatography (SEC) separation and asymmetrical flow field flow fractionation (AF4). MALS analysis of MVCs from the two orthogonal separation mechanisms helps shed light on the heterogeneity in conformation and aggregation states of each conjugate.

## 1. Introduction

**Figure 1:**
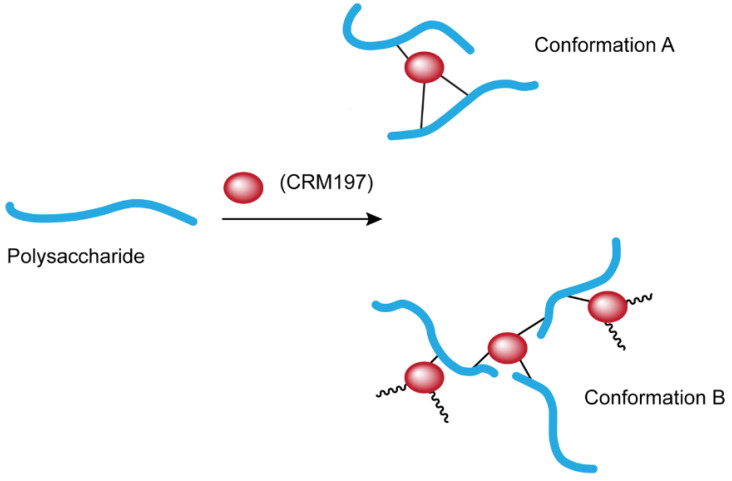
A PCV monovalent conjugate (MVC) and its conformations.

*Streptococcus pneumoniae* (*S. pneumoniae*) is a group of Gram-positive bacteria, from which infections are the major cause of community-acquired pneumonia. The major virulence factor is the exterior capsule that consists of pneumococcal capsular polysaccharide (CPS) [1,2]. Initially, multivalent pneumococcal polysaccharide vaccine (PPSV) was developed against invasive pneumococcal serotypes [3]. To boost vaccine immune response and offer better protection for infants, pneumococcal conjugate vaccines (PCVs), such as Prevnar^®^ series, Synflorix^®^ and VAXNEUVANCE™, have been developed. In the PCVs, a carrier protein, such as CRM197, is covalently conjugated to each polysaccharide (Ps) serotype (ST) to form a monovalent conjugate (MVC) (Figure 1). The resulting MVCs can recruit the T-cell dependent immune response, therefore improving the immunogenicity and enhancing immunological memory response among infants [4,5,6,7,8,9,10,11,12].

Each pneumococcal CPS is a biopolymer formed by hundreds of repeating units. The repeat unit (RU) for each CPS has a unique chemical structure that confers specific immunogenic and physicochemical properties [2]. Among more than ninety known pneumococcal CPS types, serotype 1 (ST-1) and serotype 4 (ST-4) are among the most common serotypes that can cause invasive pneumococcal diseases [13,14,15]. Furthermore, simultaneous infections by ST-1 and ST-4 dual serotypes were observed and could generate more complications in disease treatment and prevention [16]. Therefore, both ST-1 and ST-4 are included in the recent novel vaccine developments [9,11].

**Figure 2:**
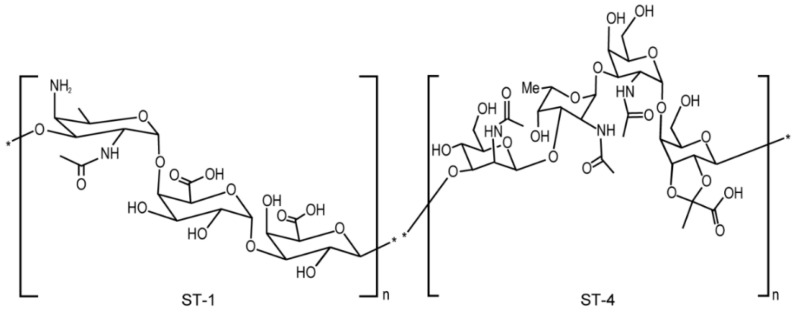
Structure of pneumococcal ST-1 and ST-4 polysaccharides.

ST-1 polysaccharide has a free primary amine and two carboxylic acid groups among three monosaccharides that form one RU. These ionic functional groups render ST-1 zwitterionic properties in solution [17,18]. ST-4 has one ionic carboxylate group among four monosaccharides within its repeating unit (Figure 2) [19]. Due to the ionic nature of the RU structures, monovalent conjugates (MVCs) generated from conjugation of a highly charged carrier protein to the poly-ionic polysaccharide are matrix-type polyelectrolyte materials. Each MVC could contain different molecular association states assembled by covalent linkages and/or non-covalent ionic interactions. A conjugate molecule can also adopt different conformations, such as the linear-like Conformation A (Figure 1) or the more branched Conformation B or/and some conformations in between. Each MVC is likely to contain conjugate molecules in a heterogenous and polydisperse nature. Molar mass (Mw) of an MVC ranges from 1–10 MDa, which is above the upper detection limit for normal mass spectroscopy. These make the physical characterization and Mw determination for such conjugated biopolymers a modern analytical challenge [20,21].

For many years, chromatographic methods have been employed to analyze vaccines and their components [21,22,23,24]. Size-exclusion chromatography (SEC) coupled with multi-angle light scattering detection (MALS) is employed as a standard method for Mw/size measurement and characterization of polysaccharide conjugates and other vaccines [25,26,27,28,29]. Generally, the SEC-MALS method offers good precision and repeatability for PCV samples in the desired concentration range. However, with increased Mw and complexity of interactions of these cross-linked protein polysaccharide conjugates, characterization by SEC could encounter unpredicted complexity. Analyte shearing degradation and pore anchoring within the SEC stationary phase have been observed and might impact the accuracy of the measurement [30].

Methods that offer gentler separation conditions or/and wider size separation range, such as asymmetric flow field-flow fractionation (AF4) or hydrodynamic chromatography (HDC), would serve as orthogonal tools to study these polydisperse high Mw polymers, in complement to the existing SEC-MALS technique [31,32,33,34]. While SEC-based separation may encounter steric interactions (gel filtration) with column stationary phases, AF4 separates/fractionates analytes are based on Brownian motion of the molecules. Therefore, generally, AF4 has a larger size range for separation, which can be beneficial for high Mw conjugate analysis. Herein, for research purposes, we have generated high Mw ST-1 and ST-4 MVCs on a small scale for SEC-MALS and AF4-MALS characterizations. Results from this study provide insight about the molecular associations and their contribution to Mw measurement.

## 2. Materials and Methods

### 2.1. Reagents and Materials

Bis-Tris-HCl 1 M solution was purchased from Rigaku Reagents, Inc. (Seattle, WA, USA). Sodium chloride 5 M solution was bought from Promega corporation (Madison, WI, USA). Bovine Serum Albumin (BSA) Standard Ampules (2.0 mg/mL) were purchased from Thermosphere (Waltham, MA, USA). The 40 kDa dextran standard was purchased from Wyatt Technology Corp. (Santa Barbara, CA, USA) and prepared as 10.0 mg/mL solution in water before use.

### 2.2. SEC-MALS Method

The SEC liquid chromatography separation was performed on an Agilent 1260 high performance liquid chromatography (HPLC) system (Agilent, DE, USA) equipped with an integrated degassing unit, a quaternary pump, a column compartment, an autosampler and a UV−Vis diode array detector. A TSKgel GMPWxL column (7.8 mm × 30 cm, 13 μm particle size, Tosoh Bioscience, Tokyo, Japan) was used for the separation condition with a 10 mM Bis-Tris, 150 mM NaCl, pH 6.8 mobile phase. An optimized flow rate of 0.8 mL/min was employed to avoid high system pressure. Column temperature was set at 35 °C and HPLC run time was 25 min for each injection.

A multi-angle light scattering detector (MALS) (DAWN^®^) and an Optilab^®^ refractive index (RI) detector (Wyatt Technology Corp., Santa Barbara, CA, USA) were connected in series to the UV-Vis diode array detector on the SEC system. In all experiments, the detectors were connected in the following order: SEC-UV-MALS-RI.

### 2.3. AF4-MALS Method

The Eclipse AF4 system was set up on an Agilent 1260 system (Agilent, DE, USA) with an integrated degassing unit, a quaternary pump, a column compartment, an autosampler and a UV−Vis diode array detector. The system was controlled by Vision^®^ software. The UV, RI and light scattering detectors were set in the same fashion as in the SEC-MALS method, with the following order: AF4-UV-MALS-RI. AF4 separation was performed on a short channel with 350 µm spacer and a 10 kDa regenerated cellulose membrane. The same forementioned Bis-Tris buffer was used as the AF4 mobile phase, at a channel flow rate of 1.0 mL/min with exponentially decreasing cross-flow gradient from 1.0 to 0 mL/min within 30 min.

### 2.4. MALS Measurement

In both SEC and AF4, the MALS detectors were calibrated using toluene and the detectors were normalized using a BSA standard according to the manufacturer’s instructions. The original retention times of the signal from different detectors are slightly different due to the sequential connections. Peak alignment and inter-detector band broadening were performed with a monodisperse BSA standard and the aligned chromatographic data were reported. All data were collected and analyzed with ASTRA^®^ software (Wyatt Technology Corp., Santa Barbara, CA, USA). The first-order fit Zimm formalism has been used as default data process procedure in ASTRA for proteins and relatively small polymers. Berry 2nd order fit was selected for the ultra-high Mw conjugates [35,36,37,38].

For each MVC, Wyatt protein conjugate analysis in ASTRA [39] was employed to deconvolute the molar mass of the protein (CRM197) from the mass of the polysaccharide. Thus, the Mw and mass for the conjugate and for the respective protein (Pr) and polysaccharide (Ps) were calculated from the software. The conjugate Mw is the sum of protein Mw and polysaccharide Mw. The UV extinction coefficient for CRM197 at 280 nm is 0.903 mL/(mg·cm) from previous internal measurements. The UV extinction coefficient of 0.667 mL/(mg·cm) was used for BSA at 280 nm based on its Certificate of Analysis provided by Wyatt Technology. A generic protein *dn*/*dc* value of 0.185 mL/g was used for CRM197 and BSA [40]. A *dn/dc* of 0.138 mL/g was provided for dextran by Wyatt Technology and used for the 40 kDa dextran standard. A literature *dn/dc* value of 0.133 mL/g was used for both pneumococcal polysaccharides [24].

The accuracy of Mw measurements was evaluated and confirmed by the measurements of a protein standard (BSA) or/and a polysaccharide (dextran) standard (Table S1, Table S2, Supplemental information). BSA has been used across different systems and columns to ensure run-to-run accuracy.

### 2.5. Monovalent Conjugate (MVC) Samples

Both ST-1 and ST-4 MVCs were produced for small-scale research and exploratory purposes by our vaccine process development group, using procedures described in the literature [28]. MVCs were directly used by both SEC and AF4 analysis without modification, unless stated otherwise.

## 3. Results and Discussion

### 3.1. SEC Measurement of High Mw MVCs

**Figure 3:**
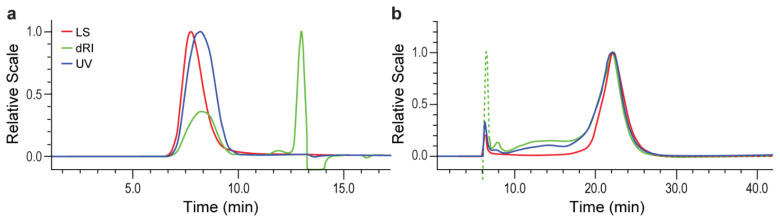
Chromatograms of a ST-1 MVC: (**a**) SEC-MALS; (**b**) AF4-MALS. (Dotted line in (**b**) represented change of channel pressure after the focus step).

The SEC method utilized was successfully demonstrated for the analysis of MVCs as in a previous report [28], and the chromatographic profile is depicted in Figure 3a. The method demonstrated good linearity, precision and accuracy during method development and optimization, and gave consistent results for most batches across various injection ranges. However, while our MVC standard showed Mw consistency across injection ranges, significant Mw increase was observed in two small exploratory conjugate batches with increased injection volume (MVC standard, ST-1 MVC and ST-4 MVC in Table 1). The measured z-average mean square radius (Rz) also varied in the same pattern, confirming the size change of the conjugate. However, such change in size was not observed for the unconjugated ST-1 and ST-4 polysaccharide precursors (Table 2).

**Figure 4:**
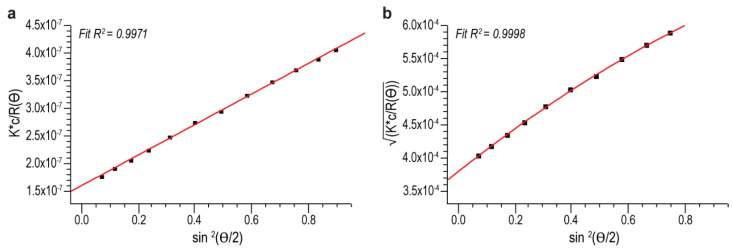
Zimm 1st order fit (**a**) vs. Berry 2nd order fit (**b**) for a ST-1 MVC on SEC-MALS. The red lines represent relationship between light scattering and angular function as described in Zimm and Berry formalisms.

**Figure 5:**
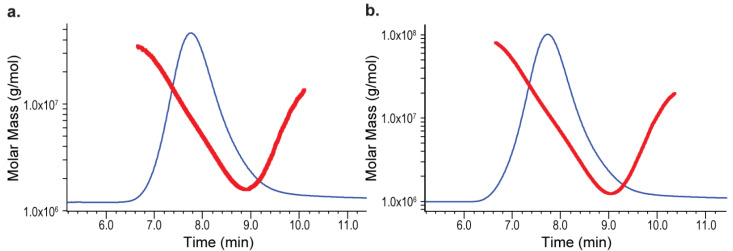
MVC Mw distribution on SEC-MALS: (**a**) ST-1 MVC; (**b**) ST-4 MVC. Each conjugate eluted out as a peak in blue trace. Conjugate molar mass distributions are shown as hook-shaped red lines.

A different formalism, 2nd order Berry, which is often preferred for high-Mw polymers [37], was also used to analyze these MVC conjugates. The MALS detector fit with both 1st order Zimm and 2nd order Berry formalisms are shown in Figure 4. Here, R^2^ (R-square) values were obtained from Astra software to evaluate the general goodness-of-fit of both formalisms, rather than the linearity of the fit. The use of 2nd order Berry formalism only reduced the variation but did not eliminate it. As seen in Table 3, the ratio of maximum Mw vs. minimum Mw (MAX/MIN) from duplicate measurements was reduced from 1.55 to 1.26 for ST-1 MVC, and from 1.73 to 1.38 for ST-4 MVC. Based on these results, we hypothesized that conjugation of the highly charged carrier protein to the charged ST-1 and ST-4 polysaccharide might have induced unexpected molecular interactions, such as self-association among the polyelectrolyte conjugate species in the MVCs. At a higher injection volume, the sample is more concentrated on the SEC column, which induces more of such self-association and results in higher Mw measured by the light scattering detector. With a lower injection volume, on the other hand, the sample is more diluted and less self-association is formed, thus a lower measured Mw. We also observed the non-ideal SEC effect from the Mw versus elution time plots depicted in Figure 5a,b. With an ideal SEC separation, Mw of a polydisperse polymer decreases with elution time. However, we observed Mw of both ST-1 and ST-4 curves up at about 9 min, a tell-tale sign of a non-ideal SEC separation, commonly observed with high Mw and branched polymers [41]. This led us to investigate these two high Mw MVCs with the orthogonal AF4-MALS measurements [42].

### 3.2. AF4 Measurement of High Mw MVCs

The AF4-MALS method was developed and optimized on a Wyatt AF4 system by applying different flow conditions and Vision Design software. The AF4 profile of the MVC is shown in Figure 3b. A Berry 2nd order fit model was used for data analysis. Both ST-1 and ST-4 MVCs were injected in duplicate at 100 µL, the standard injection volume for SEC-MALS.

**Figure 6:**
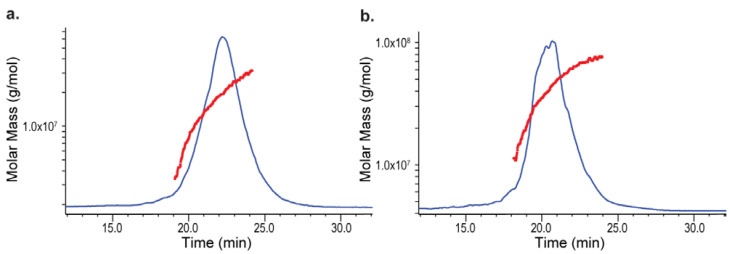
MVC Mw distribution on AF4-MALS: (**a**) ST-1 MVC; (**b**) ST-4 MVC. Each conjugate eluted out as a peak in blue trace. The Mw (molar mass) distribution across a peak was represented by a red line.

Compared to SEC, AF4 provides milder separation conditions where analytes are not trapped or/and sheared by the pores of the stationary phase beads. Indeed, Mw from AF4 increases monotonically with elution time as shown in Figure 6a,b. The data summarized in Table 4 demonstrate both Mw and Rz measured from AF4-MALS are larger than those from SEC-MALS (Table 1 and Table 3). The higher Mw measured from AF4-MALS could be attributed to not only less shearing, but also more self-association states were maintained during the milder AF4 separation. This also supported that the concentration-dependent Mw observed on SEC-MALS (Table 1 and Table 3) might be rooted from the conjugate self-association.

In addition, we also observed an unsmooth peak from ST-4 MVC sample, but not from the ST-1 MVC sample, though both were analyzed under the same conditions. The reproducibility of Mw and Rz for ST-4 is also significantly worse than that for ST-1. These observations suggest the “viscous fingering” phenomenon during the AF4 separation of ST-4. Viscous fingering happens when the sample viscosity is too high compared to the mobile phase viscosity causing non-uniform sample solution to pass through the detectors [43]. When the viscous fingering effect occurs during the separation, the apparent Mw is less reproducible and the sample peak becomes unsmooth, which are consistent with what we observed in Figure 6b and Table 4 (ST4-MVC).

### 3.3. SEC-MALS and AF4-MALS Discussion

It is worth noting that the Mw from AF4-MALS is more than two-fold higher than the corresponding value from SEC-MALS (Table 3 and Table 4). Molar mass measurements for such high Mw polyelectrolytes have been known to be challenging and are dependent on the associated/aggregated states and distribution among different populations [44,45,46,47]. Absolute Mw determination for the MVC can be complicated by the heterogeneity in Mw, self-association state and conformations. AF4 is a mild separation technique that may help maintain or/and retain the native associated states of the conjugates. Therefore, there were higher populations of associated/aggregated conjugates eluted out from the AF4 separation channel, which in turn resulted in higher Mw reported from AF4-MALS. These weakly associated reversible aggregates, on the other hand, can be broken up more under shearing inside the SEC stationary phase. The lower Mw measured from SEC-MALS may reflect more closely to the conjugate that contains less self-associated populations.

Conjugate process/formulation optimization can generate more desirable MVCs with low or no observable self-association. Our research 15-valent pneumococcal vaccine (PCV15) was formulated with fifteen optimized MVCs. Each MVC was highly diluted in the formulation buffer and bound to a proprietary aluminum adjuvant. Therefore, each conjugate in the PCV15 is believed to be free of self-interactions. If needed, such self-interaction can be evaluated by measuring the second virial coefficient (A2 or B2) of the analyte. Previously, we have reported A2 measurement for a ST-1 MVC [28].

As demonstrated here, implementing two orthogonal methods to monitor the molar mass of these complex conjugates would provide insights about key conjugate attributes and the distribution of conjugate population. This would help process development and optimization.

## 4. Conclusions

Protein-conjugated polysaccharides have been developed as potent vaccines that fight numerous deadly bacteria infections. These conjugates could exist in heterogeneous and polydisperse forms and in various association states. Accurate characterization and absolute molar mass (Mw) determination of these polysaccharide conjugates can be complicated by the self-interaction/aggregation behaviors of these highly charged species. For absolute and accurate Mw measurements, SEC-MALS has been established as a gold standard that demonstrated excellent assay performance and robustness [26,28]. It also demonstrates sensitivity to detect change in molecular association/entanglement states of a polymeric material. AF4-MALS operated under a mild separation condition may better reflect the conjugates in native states. Combined analysis from SEC-MALS and AF4-MALS would shed more light on physical states of the conjugate and offer a better understanding of Mw/size distribution and inter-molecular interaction. However, establishing a robust and precise AF4-MALS assay in quality control (QC) labs still meets many challenges, due to the need for frequent membrane change and conditioning, and the requirements of precise control on channel and cross flow rates for focusing and separation. Therefore, at this time, AF4-MALS is not suitable for commercial batch release or for quality controls. SEC-MALS remains the primary accepted assay for determination of Mw. These orthogonal analytical methods can be very useful tools for process optimization and understanding in research and development.

In all, we have presented an analytical strategy to characterize a complex polymeric product system by two complementary and orthogonal analytical methods. Such strategy should find its application in many other complex vaccine, pharmaceutical and material products.

## Figures and Tables

**Table 1:** Mw and Rz for MVCs from SEC-MALS. Zimm 1st order fit was used.

Injection Volume (µL)	MVC Standard		ST-1 MVC		ST-4 MVC	
	Mw (kDa)	Rz (nm)	Mw (kDa)	Rz (nm)	Mw (kDa)	Rz (nm)
50	4167	50	5059	140	6986	272
100	4147	51	6345	201	9713	363
150	4085	51	7863	287	12,066	419
Average	4133	51	6423	209	9588	351
MAX/MIN	1.02	1.01	1.55	2.04	1.73	1.54

**Table 2:** Mw and Rz for unconjugated polysaccharides on SEC-MALS.

Injection Volume (µL)	ST-1 Polysaccharide		ST-4 Polysaccharide	
	Mw (kDa)	Rz (nm)	Mw (kDa)	Rz (nm)
50	281	38	247	40
100	275	37	240	39
150	269	38	234	40
Average	275	38	240	39
MAX/MIN	1.05	1.04	1.06	1.03

**Table 3:** MVC Mw comparison between Zimm and Berry fit models on SEC-MALS.

MALS Fit Model	Zimm 1st Degree	Berry 2nd Degree	Zimm 1st Degree	Berry 2nd Degree
MVC Injection (µL)	ST-1 MVC Mw (kDa)		ST-4 MVC Mw (kDa)	
50	5059	4857	6986	5904
100	6345	5558	9713	7130
150	7863	6129	12,066	8159
Avg	6423	5515	9588	7064
MAX/MIN	1.55	1.26	1.73	1.38

**Table 4:** MVC Mw and Rz measured from AF4-MALS.

Injections	ST-1 MVC		ST-4 MVC	
	Mw (kDa)	Rz (nm)	Mw (kDa)	Rz (nm)
Injection-1	18,910	160	40,303	184
Injection-2	19,135	163	62,532	211
Average	19,022	161	51,418	198
MAX/MIN	1.01	1.02	1.55	1.15

## Data Availability

The datasets generated and/or analyzed during the current study are available from the corresponding author on reasonable request.

## Supplementary Materials

The following supporting information can be downloaded at: https://www.mdpi.com/article/10.3390/polym14183769/s1, Table S1: BSA Mw measured by the SEC-MALS and AF4-MALS; Table S2: 40 kDa dextran standard Mw measured by the SEC-MALS.
