# Supplementary material for: Characterization of High Molecular Weight Pneumococcal Conjugate by SEC-MALS and AF4-MALS
Source: Polymers (Basel). 2022 Sep 9;14(18):3769. doi: 10.3390/polym14183769 (PMC9501040; doi:10.3390/polym14183769)
Supplement: Supplementary file 1 [file polymers-14-03769-s001.zip › polymers-1858467-supplementary.pdf]

polymers

Supplemental information

Table S1. BSA Mw measured by the SEC-MALS and AF_4-MALS.

2.0 mg/mL BSA Injection*	Mn (kDa)	Mw (kDa)	Polydispersity (Mw/Mn)
SEC-MALS	69.3	71.8	1.04
AF4-MALS	69.7	70.1	1.01
* BSA contains small percentage of dimer are included in the analysis			

Table S2. 40 kDa dextran standard Mw measured by the SEC-MALS.

Dextran Standard	Mn (kDa)	Mw (kDa)	Polydispersity (Mw/Mn)
10 mg/mL 40 kDa Dextran	28.2	40.8	1.45
